# Perirectal abscess with dysuria

**DOI:** 10.1002/jgh3.12307

**Published:** 2020-02-06

**Authors:** Satoshi Fukuzako, Hidehito Maeda, Naohiro Koyoshi, Hiroshi Fujita, Hideyuki Kuroki, Shinichiro Uemura, Norihisa Hanada, Tadahito Urakado, Akio Ido

**Affiliations:** ^1^ Izumi General Medical Center Kagoshima Japan; ^2^ Digestive and Lifestyle Diseases Kagoshima University Graduate School of Medical and Dental Sciences Kagoshima Japan

**Keywords:** dysuria, magnetic resonance imaging, perirectal abscess

## Abstract

Perirectal abscesses often occur in the dorsal portion of the perirectal tissues. We report a patient who presented with fever, pain on defecation, and dysuria. He was found to have a perirectal abscess in the anterior perineum pressing on the urethra. After emergency surgery to drain the abscess, the symptoms improved.

## Graphical abstract

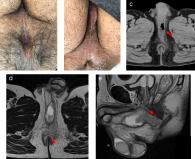

## Introduction

Perirectal and perianal abscesses are common anorectal problems. They are twice as common in men as in women, with a mean age of 40 years in both sexes.^1^, ^2^ About 90% of idiopathic perianal abscesses arise because of anal cryptoglandular infection.^3^ An abscess develops when an anal crypt gland becomes obstructed with inspissated debris, permitting bacterial growth and abscess formation. Most abscesses occur posteriorly, as 8–12 anal glands are predominantly situated around the posterior pole of the anal circumference.^4^, ^5^ An anorectal abscess should be promptly drained surgically to relieve severe pain and decrease the risk of progression to severe cellulitis or sepsis.^4^ We present a case of perirectal abscess causing dysuria due to pressure on the urethra.

## Case report

**Figure 1:**
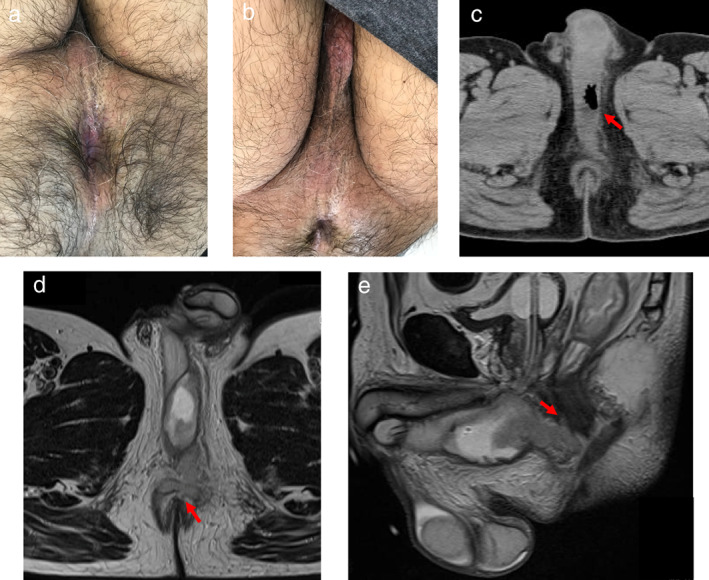
(a) No perianal redness is present, (b) but there is mild erythema of the perineum. (c) Computed tomography demonstrates a low‐density area with air in the perineum (red arrow). (d) Magnetic resonance imaging reveals continuity of the abscess with the anal canal at 12 o' clock (red arrow), (e) which is confirmed on the sagittal view (red arrow).

A 65‐year‐old man presented with a high fever, incomplete emptying of the bladder, and pain on defecation for 5 days. His temperature was 38.2°C, pulse 90 beats/min, blood pressure 120/70 mmHg, and respiratory rate 14. There was no perianal erythema, but mild redness was seen in the perineum (Fig. 1a,b). Rectal examination caused severe pain.

The white blood cell count was 19.5 × 10^9^/L. The urinalysis was within normal limits. Computed tomography (CT) showed an air‐containing low‐density area in the perineum that was pressing on the urethra (Fig. 1c). On magnetic resonance imaging (MRI), the lesion had a high‐intensity signal on a T2 weighted image and was in continuity with the anal canal at 12 o' clock (Fig. 1d,e).

We diagnosed perirectal abscess and performed emergency surgery to drain it. There was some spontaneous drainage of pus through a crypt orifice located at 12 o' clock, but no superficial fistula was present. We laid open the crypt orifice and inserted a Penrose drain. After the operation, the patient's fever, defecation pain, and dysuria improved.

## Discussion

Perianal and perirectal abscesses usually develop in the ischiorectal fossa, the intersphincteric space of the anal canal, or the rectal wall.^4^ Ischiorectal, intersphincteric, supralevator, and horseshoe abscesses are all classified as perirectal abscesses. These are usually located posteriorly because anal glands are situated near the posterior pole.^4^, ^5^ Clinically, an anorectal abscess often presents with severe pain in the anal or rectal area and fever. On physical examination, a patch of erythematous, indurated skin overlying the perianal skin may be noted in patients with a superficial abscess. A deeper abscess is often harder to diagnose and may require imaging to do so.^6^ Dysuria has not previously been reported as a symptom of perirectal abscess. In this case, upon MRI, the lesion had a high‐intensity signal on a T2 weighted image and was in continuity with the anal canal. In addition, the lesions caused urethral deformation due to extrinsic compression of the urethra. Therefore, we thought that a perirectal abscess compressed the urethra and caused dysuria. Residual urine feeling was ameliorated by draining and reducing the abscess from the crypt orifice. To the best of our knowledge, this the first report of a perirectal abscess involving the anterior perineum and causing dysuria.

Although imaging played a limited role in evaluation of perianal infections in the past, newer imaging techniques, especially MRI, are increasingly recognized as playing a crucial role in the initial evaluation.^7^ Its sensitivity is 100% and specificity 86% for detecting perianal fistulas.^8^ It is also extremely helpful for preoperative planning by outlining the extent and secondary ramifications of a fistula tract, as well as detecting anal and cutaneous openings and associated abscesses. T2‐weighted images provide good contrast between the hyperintense fluid in the fistula and the hypointense fibrous wall, allowing anatomic differentiation of the abscess from the internal and external sphincters.^9^ In this case, CT did not demonstrate continuity with the anal canal, but MRI did.

Early referral to a surgical team to discuss incision and drainage is recommended, as is avoidance of a trial of antibiotics that may delay definitive treatment. Because of the risk of deep infection, sepsis, or necrotizing soft tissue infection, patients who are immunosuppressed, have diabetes, or have evidence of sepsis or cellulitis require urgent drainage on the day of presentation.^6^ Our case illustrates the fact that a perirectal abscess may extend to the anterior perineum and cause dysuria.
